# Flexible Working, Work–Life Balance, and Gender Equality: Introduction

**DOI:** 10.1007/s11205-018-2025-x

**Published:** 2018-11-26

**Authors:** Heejung Chung, Tanja van der Lippe

**Affiliations:** 1 School of Social Policy, Sociology and Social Research, Faculty of Social Science, University of Kent, Room 106, Cornwallis Northeast, Canterbury, CT2 7NF UK; 2 Department of Sociology, Social and Behavioural Sciences, Utrecht University, Sjoerd Groenmangebouw Room C2.09, 3584 CH Utrecht, The Netherlands

**Keywords:** Flexible working, Work–life balance, Gender equality, Contexts, Organisational, Family, National

## Abstract

This special brings together innovative and multidisciplinary research (sociology, economics, and social work) using data from across Europe and the US to examine the potential flexible working has on the gender division of labour and workers’ work–life balance. Despite numerous studies on the gendered outcomes of flexible working, it is limited in that the majority is based on qualitative studies based in the US. The papers of this special issue overcome some of the limitations by examining the importance of context, namely, family, organisational and country context, examining the intersection between gender and class, and finally examining the outcomes for different types of flexible working arrangements. The introduction to this special issue provides a review of the existing literature on the gendered outcomes of flexible working on work life balance and other work and family outcomes, before presenting the key findings of the articles of this special issue. The results of the studies show that gender matters in understanding the outcomes of flexible working, but also it matters differently in different contexts. The introduction further provides policy implications drawn from the conclusions of the studies and some thoughts for future studies to consider.

## Introduction

Flexible working, that is worker’s control over when and where they work, has increased substantially over the years across most industrialised countries. Furthermore there is increasing demand for more flexibility in the workplace especially from the younger generation. Recent reports note that the majority of millennials would like the opportunity to work from home and/or have flexitime (Finn and Donovan 2013; Deloitte 2018). It is highly likely that in the future, flexible working will become the norm rather than the exception in many jobs. The question this special issue aims to examine concerns the gender discrepancies in the outcomes of flexible working for the division of labour and workers’ work–life balance. Flexible working can be used as a positive capability spanning resource useful for workers, especially women, to adapt their work to family demands (Singley and Hynes 2005). Previous studies have shown that flexible working allows mothers to maintain their working hours after childbirth (Chung and Van der Horst 2018b), and to remain in human-capital-intensive jobs in times of high family demand (Fuller and Hirsh 2018). This ability may increase women’s satisfaction with work–life balance by allowing women to maintain both. In this sense, flexible working can be a useful tool to further enhance gender equality in our societies. However, due to our society’s pre-existing views on gender roles and the gender normative views we have towards men and women’s roles and responsibilities, flexible working can potentially traditionalise gender roles in the labour market and the household (Lott and Chung 2016; Sullivan and Lewis 2001). Men use and are expected to use flexible working for performance enhancing purposes, increase their work intensity/working hours, and are rewarded more through income premiums (Lott and Chung 2016), which can increase their work–family conflict through the expansion of work. Women (are expected to) increase their responsibility within the family when working flexibly (Hilbrecht et al. 2008), which can also potentially increase their work–family conflict, but unlike men not rewarded due to the different expectations.

Although some studies already examine such gendered nature of flexible working, most are based on qualitative case studies predominately based on professional workers in the US (for example, Cech and Blair-Loy 2014). Thus we need more evidence based on large scale data, on a more representative sample from a wide range of countries and from different contexts. Country contexts matter in determining who gets access to flexible working arrangements (Chung 2017, 2018a) and in shaping the nature of flexible working (Lott 2015). National contexts can thus be expected to shape how flexible working relates to gender equality and workers’ work–life balance. Similarly, organisational contexts matter in shaping flexible working, yet is often ignored. We also need more empirical evidence encompassing larger groups of workers beyond professionals. By looking at large scale data we are able to examine how gender, class, and household structures intersect when we talk about varying outcomes of flexible working. Finally, we need to be more critical about the definitions of flexible working. Many studies conflate different types of flexible working as one, which may deter our understanding of exactly why flexible working may or may not be a useful tool in eliminating gender inequalities in the labour market.

This special issue aims to overcome these limits by bringing together innovative and multidisciplinary research (from sociology, economics, and social work) using data from across Europe and the US to address the issue of the potential flexible working has on the gender division of labour and workers’ work–life balance.

In the next section, we provide a brief overview of the existing literature to come to some of their limitations, especially in light of providing a comprehensive outlook on what flexible working can mean for gender equality. Next, we introduce the articles in the special issue and how they overcome many of the limitations mentioned previously. The introduction of this special issue finishes with a discussion, policy implications on what we can learn from these studies to ensure a better use of flexible working arrangements, and finally some notes on what is still left for us to uncover to enhance our understanding of flexible working on worker’s work-life balance and gender equality.

## Summary of Existing Literature and Their Limitations

### What is Flexible Working and the Prevalence of Flexible Working in Europe

Flexible working can entail employee’s control over when or where they work (Kelly et al. 2011; Glass and Estes 1997). More specifically, flexitime is having control over the timing of one’s work. This can entail worker’s ability to change the timing of their work (that is, to alternate the starting and ending times), and/or to change the numbers of hours worked per day or week—which can then be banked to take days off in certain circumstances. Working time autonomy, which is used in two of the papers of this special issue, is when workers have larger freedom to control their work schedule and their working hours. The biggest difference between flexitime and working time autonomy is that some constraints still remain in flexitime, in terms of adhering to core hours (e.g., 10 to 4 pm), and/or the number of hours workers can work in a day or a week (e.g. 37 h per week), unlike working time autonomy where such restrictions in many cases do not exist. Flexiplace, i.e., tele- or homework, allows workers to work outside of their normal work premises, e.g., working from home. In addition to this, flexible working can also entail workers having control over the number of hours they work, mainly referring to the reduction of hours of work (temporarily) to meet family demands. This includes part-time working, term-time only working, job sharing and temporary reduction of hours. The majority of the papers in this special issue will focus on flexitime and flexiplace, although some compare the outcomes of flexitime and flexiplace for full- and part-time workers.

**Fig. 1:**
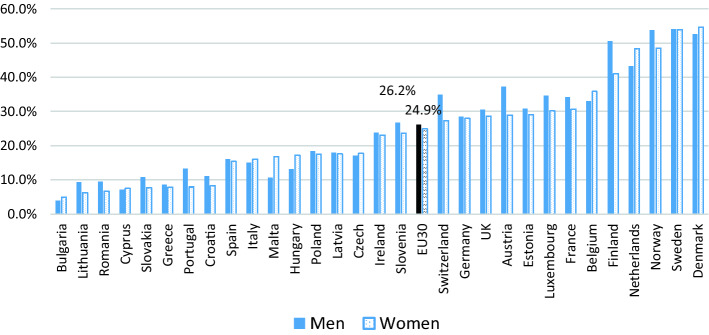
Proportion of dependent employed with schedule control across 30 European countries in 2015 (*Source*: EWCS 2015). *Note*: weighted averages/sorted by women’s %

**Fig. 2:**
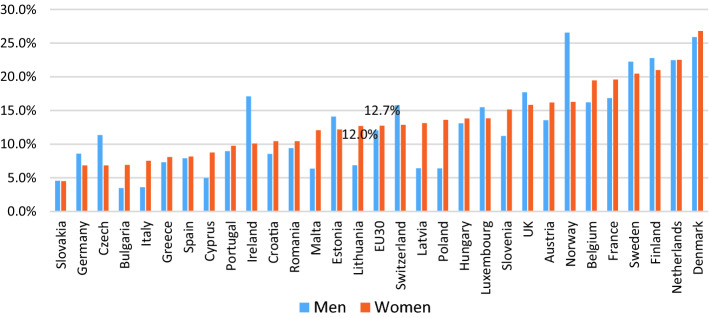
Proportion of dependent employed who work from home at least several times a month in the past 12 months across 30 European countries in 2015 (*Source*: EWCS 2015). *Note*: weighted averages/sorted by women’s %

Figures 1 and 2 provide us with the data on the extent to which flexible working is being used in Europe in 2015 based on the most recent European Working Conditions Survey. Schedule control includes workers who can adapt their working hours within certain limits (flexitime) and those with working time autonomy—i.e., where your working hours are entirely determined by yourself. Those who work from home are defined here as those who have worked in their home several times a month in the past 12 months. As we can see, about a quarter of workers had access to flexible schedules across 30 European countries and about 12% did paid work from home several times a month in the past year. There are large variations in both, where the Northern European countries are those where both schedule control and working from home are prevalent, while this is not the case in Southern and Eastern European countries. We can also see some gender differences in access/use of flexible working. At the European average the gap between men and women is not as noticeable for both schedule control and home working, although on average, men have slightly more access to schedule control while women are more likely to have worked from home. A number of countries where workers generally have more access to schedule control, it was men who were especially more likely to have access—namely, Norway, Finland, Austria, and Switzerland. However, the gender gap favourable towards men were also observed in countries with low access in general, such as Portugal, Slovakia, and Lithuania. There were only few countries where women had better access to schedule control, the Netherlands, Malta, and Hungary being some of them. For home working, with the exception of countries such as Norway, Ireland and Czech Republic, women were more likely to have worked from home regularly, or there were no discernible gender gap.

### Flexible Working and Work–Family Conflict and Gender

The relation between flexible working and work–family conflict is not as self-evident as one may expect. Of course there are several theoretical arguments to relate flexible working to less work–family conflict, and therewith higher well-being since conflict and well-being are clearly related (Back-Wiklund et al. 2011). Schedule control, that is workers’ control over when they work, provides workers with the flexibility but also control over the time boundaries between work and family spheres, enabling them to shift the time borders between work and family/care time, allowing for less conflict between the two (Clark 2000). Especially given the fact that normal fixed working hours (e.g., 9 a.m. till 5 p.m.) and family schedules/demands (e.g., school pick up times at 3 p.m.) are not necessarily compatible, control over the borders of work and home may help workers resolve some of the conflict arising from this incompatibility. Working from home allows workers to address family demands by providing a possibility to integrate the work and family domains, allowing parents to potentially combine childcare with paid work at the same time, e.g., taking care of a sick child whilst working from home. In addition, employees with long commutes are argued to have more time for childcare and/or work when they do not need to travel when they can work from home (Peters et al. 2004).

However, there is not a consistent *empirical* relation between flexible working and work–family conflict, and even less when gender is taken into account. Many studies show that working from home actually leads to *more* work–family conflict (Golden et al. 2006; Duxbury et al. 1994; Allen et al. 2013). Control over when to work in addition to working from home is also only partly related to less work–family conflict (Michel et al. 2011).

Still, there are studies that provide evidence that flexible working relieves work-to-family conflict (e.g., Allen et al. 2013; Kelly et al. 2014; Michel et al. 2011) especially during the transition into parenthood (Erickson et al. 2010). Ten Brummelhuis and Van der Lippe (2010) reported that employees’ family situation matters, and that working from home and flexible work schedules were only effective in relieving work–family conflict for singles and not for employees with a partner and/or children. Demerouti et al. (2014) argue in their overview study on the impact of new ways of working, including working from home and flexible schedules, that these mixed findings for work–family balance and conflict are not surprising. Due to the fact that the permeability of boundaries between work and nonwork domains increases when workers work flexibly, as physical boundaries between the two environments are eliminated. Instead of facilitating balance, flexible working can thus also lead to increased multitasking and boundary blurring (Schieman and Young 2010; Glavin and Schieman 2012).

The relationship between flexible working and work–family conflict have different outcomes for men and women, as women are often still more responsible for housework and childcare and spend more time on these chores (Van der Lippe et al. 2018; and also see the next section). The effect of work role ambiguity on work–family conflict is also different for men and women (Michel et al. 2011). Moreover, different arrangements may have different outcomes for men and women. Peters et al. (2009) showed that female workers gained better work life balance from more control over their work schedule leading to a better work–family balance. However, home-based teleworking|women did not experience a better work–life balance than employees not working from home. Nevertheless, there are only a few studies where, in a systematic and rigorous way, the differences between men and women are studied, and most results rely on qualitative studies (Emslie and Hunt 2009). Most studies are also constrained by the gender neutral assumption of work–life balance (see for an excellent overview, Lewis et al. 2007). The next section explores further why this is the case.

### Flexible Working and the Expansion of Work and Domestic Spheres and Gender

One of the reasons why flexible working may not reduce work–family conflict of workers is because it is likely to lead to an expansion of work and/or increase the domestic burden upon workers.

Unlike what many studies that look at flexible working as a family-friendly arrangement would assume, flexible working have been shown to result in the expansion of the work sphere rather than the contraction of it, resulting in paid work encroaching on family life (Glass and Noonan 2016; Lott and Chung 2016; Kelliher and Anderson 2010; Schieman and Young 2010). Several theories can explain why such expansion occur (see for more detailed theories, Kelliher and Anderson 2010; Chung and Van der Horst 2018a; Lott 2018) but this can be summarised into gift exchange—workers feeling a need to reciprocate for the gift of flexibility back to employers; enabled intensification—blurring of boundaries allowing workers to work harder/longer than they otherwise would have; or enforced intensification where employers may increase workload alongside providing workers more flexibility over their work.

Clark (2000) argues that the flexibility between the borders of the work and home domain will result in different outcomes, for example, expansion of one sphere and the contraction of others, depending on the strength of the border, the domain the individual identifies with most, and the priority each domain takes in one’s life. In other words, for those who prioritise paid work above home and other aspects of their life, the flexibility in the border is more likely to result in the expansion of paid work, while for those whose priorities lie in the home spheres, flexibility may result in the expansion of domestic activities, such as housework and care giving. One important point to raise here, is that it isn’t necessarily an individual’s choice to prioritise paid work or home spheres, and external demands and social norms shape one’s capacities to do so.

The ability to prioritise work and adhere to the ideal worker culture, that is a worker that has no other obligation outside of work and privileges work above everything else, is gendered (Acker 1990; Williams 1999; Blair-Loy 2009). Although there have been some developments, men still do and are expect to take on the breadwinning role especially after childbirth (Miani and Hoorens 2014; Knight and Brinton 2017; Scott and Clery 2013) and women are thus left to and are expected to take the bulk of caregiving for both children and ill relatives as well as housework (Hochschild and Machung 2003; Bianchi et al. 2012; Hook 2006; Dotti Sani and Treas 2016). Such gendered divisions of labour and social normative views about women and men, and more specifically mothers’ and fathers’ roles shape how flexible working is performed and viewed by society, including employers but also colleagues, friends, families etc., and consequently on the outcomes of flexible working.

It is true that previous studies that examined the gender discrepancies in the expansion of working hours, more specifically overtime hours, due to flexible working find that men are more likely to expand their working hours than women (Glass and Noonan 2016; Lott and Chung 2016).

On the other hand, flexible working is likely to be used by women for caregiving purposes (Singley and Hynes 2005) and those who do work flexibly are likely to expand their care/housework (Sullivan and Lewis 2001; Hilbrecht et al. 2013). Clawson and Gerstel (2014) argue that, flexible working allows workers—especially middle class workers, to ‘do gender’ (West and Zimmerman 1987) in that they are able to fulfil the social normative roles prescribed within societies. This then feeds into what people believe flexible working will result in for men and women. For example, qualitative studies have shown that when women take up flexible working arrangement, for example working from home, those around them expect women to carry out domestic work simultaneously whilst working (Sullivan and Lewis 2001; Hilbrecht et al. 2013; Shaw et al. 2003). This consequently shapes how people provide and reward/stigmatise flexible working of men and women. Lott and Chung (2016) using longitudinal data from Germany show how even when women work longer overtime when taking up flexible schedules, they are still less likely compared to men to gain any financial premiums. Furthermore, mothers seem to be exchanging the opportunity to work flexibly with longer overtime, i.e. not even gaining an ‘overtime premium’ for the additional hours worked. Similarly, several recent experimental studies based in the US have shown that women, especially mothers, are less likely to gain access to flexible working arrangements, even when not used for care purposes, and more likely to be stigmatised for its use compared to men (Brescoll et al. 2013; Munsch 2016). For fathers, on the other hand, there seems to be a “progressive badge of merit” (Gerstel and Clawson 2018) where they are generally looked favourably upon for using flexible working arrangements for care purposes. Again this is largely down to the expectations people hold regarding how men and women will use their flexibility. In other words, in countries where traditional gender norms are prevalent, even when fathers take up flexible working for care purposes, there is a general expectation that the fathers will still maintain their work devotion/protect their work spheres and prioritise it over family time/care roles. On the other hand, for mothers, people expect them to use their control over their work for care purposes, even when it is explicitly requested for other more performance enhancing purposes. This can explain why flexible working arrangements that provide workers more control over their work are less likely to be provided in female dominated workplaces (Chung 2018a, c).

Such preconceived notions of where worker’s priority lies and how they will use the increased control over their work will naturally shape the consequences of flexible working for one’s career. Leslie et al. (2012) show how flexible working for performance enhancing purposes is likely to be rewarded, while that for family-friendly purposes will not. Williams et al. (2013) provide evidence on how flexible working for family purposes can actually lead to negative career consequences, again largely due to the fact that  flexible working for family purposes makes workers deviate away from the ideal worker image. In this sense, flexible working can potentially increase gender inequalities in the labour market, due to the preconceived notion people will make about women’s flexible working. However, this is not always the case. Several studies have shown that flexible working may allow women to work longer hours than they would have otherwise after childbirth (Chung and Van der Horst 2018b) and stay in relatively stressful yet high paying occupations (Fuller and Hirsh 2018) and workplaces with flexible working arrangements are those where the gender wage gap is smaller (Van der Lippe et al. 2018). Thus the picture is rather complex in terms of what flexible working can mean for gender equality.

## About the Special Issue: Addressing the Gaps in the Literature

Despite the large number of studies that deal with flexible working and the nuanced gendered ways in which it may mean different things for men and women, there are some limitations which the papers of this special issue will try to overcome.

One of the biggest limitations of previous studies on this topic is that they are mostly based on qualitative data—mostly interviews and observations. In addition, many of the studies also focus on professionals. Although there have been some studies using quantitative time use data (Craig and Powell 2011, 2012; Wight et al. 2008) most have been using data from Anglo-Saxon countries, namely US, UK and Australia. Given that work cultures as well as gender norms are expected to heavily shape the way in which people perceive how workers will use flexibility in their work, and how workers perform flexibility, we need more evidence from a broader range of countries to be able to understand how flexible working can lead to different outcomes for men and women.

### Role of Contexts

Investigating the role of contexts is the core of the contribution from Kurowska (2018). Here the main aim is to examine the gender differences in how working from home deters or enhances one’s work life balance comparing dual earner couples in Sweden and Poland, two very different countries in terms of their gender relations and family policy support. Sweden is well known to be a country with gender egalitarian norms, generous family policies including ear-marked paternity leaves that promote fathers’ involvement in childcare. Poland is known as a typical conservative/traditional care regime, where mothers (are expected to) take on the bulk of care roles of children. Another unique contribution of this paper is its use of the theoretical concept, ‘total burden of responsibilities’ to capture the engagement in both unpaid domestic work responsibilities in addition to one’s time spent on paid work, to provide the capability of an individual to balance work with leisure. She finds that men in both countries have higher capabilities to balance work with leisure than women, but the difference between genders is smaller in Sweden than in Poland. She further finds that working from home is related to lower capability to balance work with leisure for mothers in both countries, while this is not the case for fathers in Poland. The results of this study show how gender norms of the country, and the respective expectations towards mothers and fathers shape the extent to which flexible working can lead to increasing or decreasing the gender gap in domestic work.

The importance of context does not only lie at the country level. One main area most studies fail to incorporate is the extent to which organisational level contexts matter in shaping how flexible working relate to different work–family outcomes for men and women. Van der Lippe and Lippényi’s (2018) paper aims to tackle this issue in more depth. Their main contribution is to examine how organisational culture and context can play a role in the way working from home may reduce or exacerbate one’s work-to-family conflict for men and women. Here organisational contexts include supportive and family-friendly organisational culture as well as the normalisation of flexible working, as indicated by the number of colleagues working from home. These organisational contexts are expected to moderate the relation between working from home and work–family conflict. Using the unique dataset European Sustainable Workforce Survey, they are able to compare workers from across 883 teams, in 259 organisations, across nine countries (Bulgaria, Finland, Germany, Hungary, Netherlands, Portugal, Spain, Sweden, UK). Results show that working from home leads to more work–family conflict, especially when workers perceive an ideal worker culture at their workplace and less so when there are more colleagues working from home. The influence of culture seems to be more important for women than men, for whom work culture matters less.

These studies shine an important light on how the importance of the context in which flexible working is used matters in determining not only its outcome but also the gender discrepancy in the outcomes.

### Defining Flexible Working, Discrepancies Between Arrangements

Another limitation of previous research is the way flexible working is operationalised. Many studies do not distinguish between different types of flexible working, in the extent to which control is given, and for which purpose.

Lott (2018) aims to tackle this issue by distinguishing between the different types of flexible schedules to see how they relate to work-to-home spill-over for men and women. Using the German Socio-Economic Panel Study in 2011 and 2012, she distinguishes between three different types of working time arrangements. Namely, she distinguishes between flexitime—i.e., a certain degree of self-determination of daily working hours within a working time account, and working-time autonomy—no formally fixed working hours and where workers choose their own working hours, and for the lack of control, fixed schedules against employer-oriented flexible schedules—namely, working hours fixed by employer, which may vary from day to day. She finds that employees experience the most work-to-home spillover with working-time autonomy and employer-oriented schedules, and the least with flexitime and fixed schedules. However, she also finds gender differences. Working-time autonomy’s association with higher cognitive work-to-home spillover only holds for men, and mainly due to the increased overtime hours men work when having working-time autonomy. Another unique contribution of this paper is the inclusion of employer-oriented flexible schedule—i.e. how unpredictability and unreliable schedules influence work–life balance. Here she finds that such unpredictability and unreliability is especially problematic for women; only women seem to suffer from higher spillover with employer-oriented schedules. This relationship holds above and beyond job pressure and overtime hours. Lott argues that the main cause for this is due to women’s position as the main person responsible for the day to day management of the household, for which such unpredictability of working hours can be extremely problematic. For similar reasons women seem to suffer less with flexitime—in that they have more control over their schedules.

Chung and Van der Horst’s (2018a) study also aims to distinguish between different types of flexible working arrangements—namely schedule control, flexitime, and teleworking. One of the main contribution of their study is to distinguish between workers’ control over their working hours, but for different purposes—namely those primarily used for family-friendly goals (flexitime), against those provided mostly for performance enhancing goals (here for convenience referred to as “schedule control”). They examine how these different types of workers’ control over their work are associated with an increase in unpaid overtime hours of workers for men and women in the UK using the Understanding Society data from 2010 to 2015 and fixed effects panel regression models. Results show that flexitime and teleworking do not increase unpaid overtime hours significantly. On the other hand, the more performance enhancing schedule control increases unpaid overtime hours, but with variations across different populations. Unsurprisingly, mothers, especially those working full-time, appear to be less able to increase their unpaid overtime as much as other groups of the population. This can be mostly explained through the fact that many mothers working full time would not have any more time to give to their companies, unlike many men, including fathers, and women without children. On the other hand, part-time working mothers increased their unpaid overtime hours significantly when using schedule control. This discrepancy in the ability to work longer hours can potentially increase gender inequality in the labour market due to overtime being seen as one of the most explicit forms of commitment towards the company. Yet in the case of part-time working mothers, it is unlikely that these increased hours will result in additional career premiums as evidenced in another contribution of the special issue (Chung 2018c).

Chung (2018c) distinguishes between flexitime, working from home, and part-time work when examining workers’ experiences with flexibility stigma, that is the negative perception towards those who work flexibly, using the 4th wave of the Work–Life Balance Survey conducted in 2011 in the UK. She finds that men are more likely to agree with the statement that those who work flexibly generate more work for others, and say that they themselves have experienced negative outcomes due to co-workers working flexibly. On the other hand, women and especially mothers are likely to agree that those who work flexibly have lower chances for promotion and say they experienced negative career consequences due to themselves working flexibly. One reason behind mother’s experience with flexibility stigma is due to the fact that most mothers use some sort of working time reducing arrangement, e.g. part-time work. On the other hand, men and fathers are more likely to use flexitime and teleworking, which are less likely to lead to negative career outcomes. Chung further argues that it might be simplistic to completely attribute the differences found between men and women in the negative career outcomes experienced when working flexibly, only to the types of arrangements they use. In other words, the negative career outcomes experienced by part-time workers may partly have to do with the fact that it is widely used by mothers to balance work with family life (see also, Lewis and Humbert 2010). Thus, the stigma towards part-time workers’ commitment towards work and productivity may be better understood as a reflection of the stigma towards mothers’ commitment towards work and their productivity.

Kim (2018) examines how flexible working policies increase parental involvement with children and also distinguishes between different types of flexible working policies, namely access to flexitime/flexible schedules, ability to work at home, and working part-time. Using the longitudinal data from the US Early Childhood Longitudinal Survey-Birth Cohort (ECLS-B), he finds that working from home was associated with more frequent enrichment parent–child interactions, but only for mothers, echoing what was found in Poland by Kurowska (2018). Part-time working for mothers was also associated with more frequent enrichment parent–child interactions, and for father’s access to flexitime were associated with greater daily routine interactions. The result of increased routine care of fathers through flexitime is most likely due to tag-team parenting (Craig and Powell 2012) where parents use flexible schedules to increase the time both parents spend with children. By enabling men to take up a larger share of routine care of children flexitime of male partners can help women build their careers—which explains why men’s flexitime has been shown to increase women’s career perspectives (Langner 2018).

These studies provide us with evidence that we need to look at the intersection between gender and different types of flexible working to better understand how flexible working leads to different outcomes. Furthermore, they enable a better understanding of how different types of flexible working may result in different outcomes for gender equality. Working from home, working time autonomy/schedule control for performance purposes may not necessarily provide much benefit to even out the playing fields for men and women. On the other hand, flexitime—especially with a more defined/clear working hours boundaries, seems to be a better option if we are to ensure flexible working does not lead to further traditionalisation of gender roles.

### Incorporation of Class

Another contribution the papers in this special issue is to examine the intersection between gender and class when examining the outcomes of flexible working. Many of the existing studies on flexible working focus on professionals (e.g., Cech and Blair-Loy 2014), which to some extent relate to the access these groups have towards flexible working arrangements and control over their work (Chung 2018a). However, the intersection between gender and class has been shown to be of great importance in understanding how flexible working enables workers to do or undo gender (Clawson and Gerstel 2014; Deutsch 2007). The articles in this special issue also try to engage in the analysis of class, to see how there may be distinctions between classes in the way flexible working relate to gendered outcomes.

Kim (2018) in his analysis of how flexible working may lead to different levels of parent–child interactions, incorporates household structures and income as well as gender. The results indicate that the positive impacts of flexible working vary depending on income levels and for single/dual earner households. For example, the positive association between working from home and parent–child interactions was more pronounced among low-income mothers than mid- and high-income mothers. Part-time working only increased enrichment interactions with children for mothers in two-parent families, perhaps reflecting the limited capacity of single-mothers to expand their time on such activities. Part-time working increases parent–child interactions only for fathers from dual-earner households and not for those from single-earner households. This finding reflects the results found in previous studies regarding gender division of labour within households of female-breadwinner families (Bittman et al. 2003).

By examining the lack of schedule control, Lott (2018) also focussed on the less privileged, mostly non-professional, lower-class workers whose work schedule are more often determined by the employer and changed on a daily basis. She found that work–life spill-over is highest for these workers, especially women. Women of the lower working class have fewer financial resources in order to cope with unpredictable and unreliable work hours, for example to pay for public or private childcare. They alone carry the double burden of balancing paid and unpaid work.

Chung and Van der Horst (2018a) examine the differences between different occupational groups in their analysis of how flexible working leads to increased unpaid overtime hours for men and women, parents and non-parents. They find that the increase in unpaid overtime hours when workers have control over their schedule was largely driven by the professionals in the model, especially for men. In closer inspection, there seems to be a division within professionals in terms of gender when we consider parenthood. Professional men with and without children seem to increase their unpaid overtime hours especially when they have a lot of schedule control, while professional women with children do not. On the other hand, professional women without children increase their overtime hours similar to that of men, yet again it is questionable whether they will benefit from the same career premium from it (Lott and Chung 2016).

## Discussion, and Policy Implications and Future Challenges

The results of the papers in this special issue point to one conclusion; flexible working can be useful in enabling a better work–life balance and family functioning, yet we need to be aware of the potential gendered ways in which it is being/and is expected to be used. In other words *gender matters* when it comes to understanding the consequences of flexible working. Men and women use flexible working in different ways that leads to different outcomes for wellbeing, work–life balance and work intensification. A recurring finding is that women are more likely to (or expected to) carry out more domestic responsibilities whilst working flexibly, while men are more likely to (or are expected to) prioritise and expand their work spheres. Consequently, it is women who will fear and are more likely to face negative career outcomes due to flexible working as Chung (2018c) shows. However, we need to be careful about understanding such patterns as a matter of choice. As Lott (2018) has argued, family and domestic responsibilities may be understood more as a constraint under which women need to navigate and negotiate their work spheres.

Furthermore, we must also conclude that gender is a too general distinction to gain insight in the consequences of flexible working on work–life balance outcomes. A common thread found in all articles in this special issue is that gender must be studied in context; in the organisational, country, family, as well as class context. First of all, the culture of the organisation matters, such as the prevalence of flexible working in the organisation as well as supervisory support etc., yet perhaps more for women as Van der Lippe and Lippényi (2018) show. Second, country contexts matter in that flexible working allows workers to “do gender” in a more traditional gender cultures such as Poland, and where a more gender egalitarian culture exists, such as in Sweden, the gender discrepancies due to flexible working may not be as evident, as Kurowska (2018) shows. Third, the household structures appears to be important in the outcomes of flexible working. There are differences in single versus dual earners, as well as low- versus higher income families for both men and women as Kim (2018) shows us. The occupation of the worker also matters, where the gender discrepancies in the negative spill-over effects, namely working long unpaid overtime hours, of schedule control depend on the occupation you look at as Chung and Van der Horst (2018a) show. Overall, the findings in this special issue seem to indicate that especially in contexts where traditional norms on gender roles are prevalent and where ideal worker culture exists, flexible working may promote a more traditionalised division of labour resulting in hindering rather than supporting gender equality. This is likely because in such contexts, flexible working can lead to women being able to (but also having to) expand their household burdens, while men expand their work loads. This may reinforce the (unconscious) biases employers and co-workers have towards flexible working of men and women, and more female oriented and male oriented flexible working arrangements, which can increase the wage gap between the genders as Chung’s (2018c) work indicates.

So what can be done to prevent such increase in traditionalisation through flexible working? At the macro level, there needs to be changes in our gender norms and ideal working culture. In other words, flexible working is not used in a vacuum and as long as our gender normative views about mothers and fathers roles do not change, the way people perceive flexible working will be used by men and women is unlikely to change and will feed into how they will in fact be used. Attention is required, for example via the European Institute for Gender Equality (EIGE) at the European level, but also other national level bodies for promotion of gender equality in Europe and its member states through delivering expertise and knowledge, and enhancing policies to change normative views of gender roles. Policy changes, such as increase in well paid ear-marked paternity leaves, such as the ones found in Sweden, has been shown to increase father’s involvement in childcare and domestic work not only in the period during the leave but many years after (Nepomnyaschy and Waldfogel 2007). Thus it can be used as a useful tool to help reduce the gender division in childcare and household tasks, and consequently help shift the gender norms of the country. Consequently such policies can also be useful in ensuring that flexible working is not used as a tool to enforce traditional gender roles. Providing better protective mechanisms for workers to ensure that flexible working and blurring of boundaries do not lead to encroachment of family life would also be important policies to be implemented at the national level. Current labour laws, which is based on a more traditional 9 to 5 job done in the office, may not be sufficient to ensure such protections.

One key finding of our research was that when flexible working becomes more of a norm, rather than the exception, this may help workers use flexible working arrangements for work–life balance purposes. Changing the right to request flexible working legislations to ensure that flexible working is more of a right from day 1 on the job, that flexible working is more of a default rather than an exception would be useful in ensuring that it does not lead to stigma or work–life conflict.

At the mezzo and micro level, we need to make sure both workers and managers are aware of the risks of flexible working. For companies, providing good role models of senior managers, especially male senior managers, taking up flexible working for family-purposes and without work spilling over to other spheres of life will be important to show how best to utilise flexible working. The notion of ‘the healthy organisation’ might be helpful here. Healthy organisations take into account the wellbeing and work family balance of employees, as well as workplace effectiveness (Lewis et al. 2011). Building better collective practices of flexible working, where work is not done everywhere and all the time, is crucial. It implies that organisations implement flexible work options under the condition that it is rewarding (in a material and unmaterial way) for employees and such that it commensurate with it success (Lewis et al. 2011). Workers themselves should also be reflective of how some of their own expectations in how flexible working should and can be used is shaped by our prevailing gender norms and assumptions on whose job it is to care/do the breadwinning. To question some of the gendered assumption would be important.

One of the challenges is how to take the family situation better into account when implementing work flexibility in such a way that it enhances work–life balance. One of the ways could be to relieve work and household burden, often a double burden for women when they also have a paid job (Hochschild and Machung 2003). Arrangements for example regulating working hours and applying flexible time-management models suited to the needs of the employee and his or her family. Other options are a professional network of family support services, including public childcare, elderly care services, different forms of leaves, as well as arrangements to outsource housework (De Ruijter and Van der Lippe 2007). Of course a discussion is needed who is responsible for these arrangements and to what extent. Is it the individual employee, the country individuals live in, or the organisation of the employee? Most likely this will be a combination of all three, also partly dependent on the welfare regime of the country, and the sector the organisation of the employee belongs to. Public policies and interventions are for example deeply embedded in Scandinavian culture. They may fit less with the cultures, habits and structures of other European welfare states, but organisations might take the lead more in these welfare states.

There are some issues that this special issue has not been able to address. Firstly, we still know very little about how flexible working relate to informal care capacities. Majority existing studies, including the ones in this special issue, deal with flexible working for childcare purposes. More research is thus needed to see how flexible working is gendered (or not) in increasing workers’ care capacities in times when informal care demands arise, or how it allows workers to combine work with informal care demands. Secondly, longer career consequences of flexible working, especially relating to flexitime and tele/home working, would be useful to investigate, especially in order to understand how flexible working relate to gender wage gaps. Some of the studies here and  other previous studies have shown that flexible working can increase men's working hours/overtime hours and other commitment towards work which may increase their wage premiums, and consequently the gender wage gap between men and women. On the other hand, flexible working also helps women reduce work family conflict and allow them to work longer than they would've otherwise. In this sense, exactly how these two rather conflicting dynamics add up in the longer run would be important to examine. Thirdly, more analysis is needed to fully understand the importance of context in not only shaping the outcomes of flexible working, but also how it shapes the gendered nature of flexible working. Our studies have shown that gender norms and long hours cultures have been shown to be important contexts that shape such outcomes. Examining these and other contextual factors, such as the strength of the legal right to flexible working, its prevelance, and workers’ negotiation power, both at the national and organisational levels will help us find out more about under which context can we expect a better use of flexible working so that it enhances both workers' work life balance and gender equality. We hope that this special issue has provide some useful steps in the right direction to find these answers out, and that it helps pave the way for future scholars to follow. Flexible working is likely to become more common in the future as demands for flexible working increases among both new and older generations of workers for diverse reasons. It provides us with great opportunities to tackle some of societies’ most pressing challenges. However, as this special issue has shown, this will only be the case if it used in the right way. 
